# Sonic hedgehog signaling in kidney fibrosis: a master communicator

**DOI:** 10.1007/s11427-016-0020-y

**Published:** 2016-06-22

**Authors:** Dong Zhou, Roderick J. Tan, Youhua Liu

**Affiliations:** 1 Department of Pathology, University of Pittsburgh School of Medicine, Pittsburgh, Pennsylvania 15261, USA; 2 Department of Medicine, University of Pittsburgh School of Medicine, Pittsburgh, Pennsylvania 15261, USA; 3 State Key Laboratory of Organ Failure Research, National Clinical Research Center of Kidney Disease, Nanfang Hospital, Southern Medical University, Guangzhou 510515, China

**Keywords:** Sonic hedgehog, Gli, tubular cells, fibroblast, renal fibrosis

## Abstract

The hedgehog signaling cascade is an evolutionarily conserved pathway that regulates multiple aspects of embryonic development and plays a decisive role in tissue homeostasis. As the best studied member of three hedgehog ligands, sonic hedgehog (Shh) is known to be associated with kidney development and tissue repair after various insults. Recent studies uncover an intrinsic link between dysregulated Shh signaling and renal fibrogenesis. In various types of chronic kidney disease (CKD), Shh is upregulated specifically in renal tubular epithelium but targets interstitial fibroblasts, thereby mediating a dynamic epithelial-mesenchymal communication (EMC). Tubule-derived Shh acts as a growth factor for interstitial fibroblasts and controls a hierarchy of fibrosis-related genes, which lead to the excessive deposition of extracellular matrix in renal interstitium. In this review, we recapitulate the principle of Shh signaling, its activation and regulation in a variety of kidney diseases. We also discuss the potential mechanisms by which Shh promotes renal fibrosis and assess the efficacy of blocking this signaling in preclinical settings. Continuing these lines of investigations will provide novel opportunities for designing effective therapies to improve CKD prognosis in patients.

## INTRODUCTION

It is estimated that the prevalence of chronic kidney disease (CKD) is about 10%–12% of the adult population worldwide (Coresh et al., 2007; Zhang et al., 2012; Zoccali et al., 2010). As a general rule, tissue fibrosis develops in virtually every form of CKD, as both a consequence and a characteristic feature of disease progression. From a cell biological standpoint, the intricate process of renal fibrosis has been linked, more or less, to an inappropriate activation of some key signaling pathways, such as transforming growth factor-β (TGF-β), renin-angiotensin system (RAS), Wnt/β-catenin, and sonic hedgehog (Shh) (He and Dai, 2015; Meng et al., 2015; Santos et al., 2012; Sweetwyne et al., 2014; Tan et al., 2014). Most of the time, these diverse signals reciprocally crosstalk through cell-to-cell communication that involves all resident cells in the kidney, as well as the infiltrating cells (Duffield, 2014; Liu, 2011; Zeisberg and Neilson, 2010). In the past several years, the role and mechanisms of TGF-β, RAS and Wnt/β-catenin signaling in the pathogenesis of CKD have been well documented (Meng et al., 2015; Sweetwyne et al., 2014; Tan et al., 2014; Zhou et al., 2015). Therefore, the scope of the present review is focused on our current understanding of Shh signaling in renal fibrogenesis.

Hedgehog signaling is an evolutionarily conserved, developmental pathway that plays an essential role in regulating mammalian embryonic development (Robbins et al., 2012). Hedgehog was identified in genetic screens in *Drosophila* in 1980, and the vertebrate hedgehog proteins were discovered in 1993 (Krauss et al., 1993; Nusslein-Volhard and Wieschaus, 1980). Since then, investigations on the role of hedgehog signaling in organ development and diseases progression have rapidly accelerated. There are three hedgehog ligands in mammals: Sonic hedgehog (Shh), Indian hedgehog (Ihh) and Desert hedgehog (Dhh), of which Shh is the best characterized (Lum and Beachy, 2004). Accumulating studies have demonstrated an activated Shh signaling in fibrotic CKD, suggesting a potential connection between aberrant regulation of this signaling and kidney fibrosis (Ding et al., 2012; Fabian et al., 2012; Kramann et al., 2015; Rauhauser et al., 2015; Zhou et al., 2014).

In this review, we will summarize the key features of Shh signaling and its regulation in various forms of CKD, and discuss the targets and the modes of action of Shh, as well as its relevant signal transduction routes. We will also provide the perspectives of several strategies to intervene Shh signaling for an effective therapy of the patients with fibrotic CKD.

## SONIC HEDGEHOG SIGNALING: COMPONENTS, ROUTES AND MECHANISM

### Shh ligand

As a morphogen, Shh is important in tissue patterning during embryonic development by controlling multiple biological processes including cell fate determination, cell proliferation and differentiation (Gill and Rosenblum, 2006; Pan et al., 2013). Human Shh is synthesized as a 45 kD precursor protein with 462 amino acids, in which the first 23 amino acids serves as a signal peptide. It is autocatalytically cleaved to produce a 19 kD amino-terminal fragment (N-Shh) and a 25 kD carboxyl-terminal domain (C-Shh), and then secreted into the extracellular space. N-Shh retains all known signaling capabilities, while C-Shh possesses protease activity.

**Figure 1:**
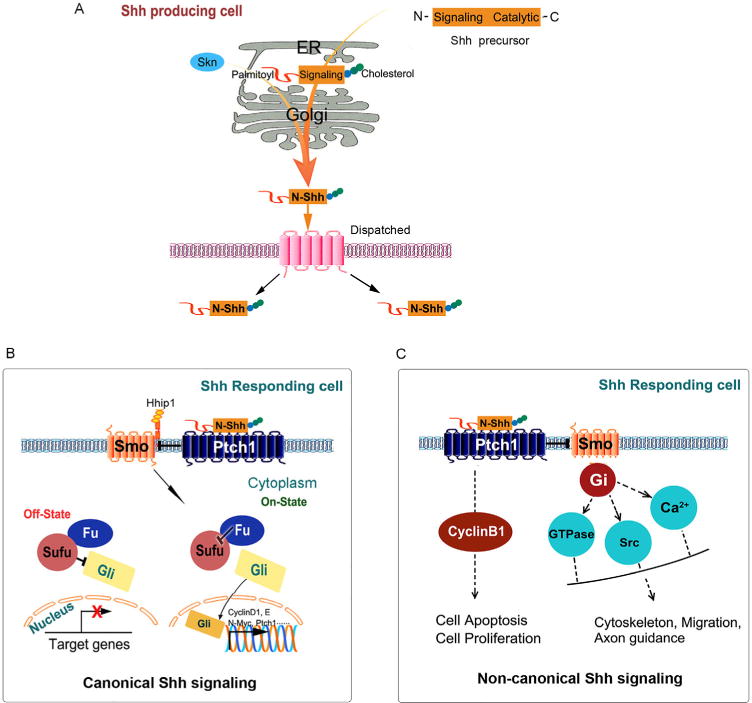
Schematic illustration of the Shh signaling pathway. A, In Shh-producing cells, the Shh precursor is proteolytically cleaved to generate the N-Shh in the endoplasmic reticulum (ER). Secreted Shh contains two covalent cholesterol modifications: a C-terminal cholesterol moiety and a palmitoyl group is added at the N-terminus. Skn and Dispatched, mediate the release of Shh ligand. B, The canonical Shh signaling. In the inactive state, the transmembrane protein receptor Ptch1 interacts with and inhibits the activity of a seven transmembrane protein, Smo. Interactions with cytoplasmic proteins, including Fused and Sufu, the transcription factors Gli are prevented from entering the nucleus and downstream target genes expression are repressed. In the active state, Shh binding to Ptch1, which allows Smo activation, thereby activating the cascade that leads to the Gli transcription factors to exert their effects in the nucleus. C, The non-canonical Shh signaling. There are two types of non-canonical Shh signaling pathways, one is Ptch1-dependent which regulates cell apoptosis and proliferation, the second is Smo-dependent which associate with modulation of actin cytoskeleton-dependent processes. Shh, Sonic hedgehog. N-Shh, N-terminal Sonic hedgehog. Skn, Skinny hedgehog. Ptch1, Patched-1. Smo, Smoothened. Hhip1, Hedgehog-interacting protein 1. Fu, Fused. Sufu, Suppressor of fused. Gli, Glioma-associated oncogenes.

During the cleavage, a cholesterol molecule is added to the carboxyl end of the N-terminal domain, which is involved in trafficking, secretion and receptor interaction of the Shh ligand (Figure 1A). Typically, secreted Shh contains two covalent modifications, a C-terminal cholesterol moiety and a palmitoyl group to the N-terminal of the processed N-Shh. Despite its dual lipid modification and tight association with cell membranes, the Shh protein acts directly on distant cells in developing tissues. In vertebrates, this remote action requires the transmembrane transporter-like protein Dispatched (Disp) and Skinny hedgehog (Skn), which mediate the release of Shh from secreting cells (Lum and Beachy, 2004). Shh elicits its biological activity via both an autocrine and paracrine fashion.

### Canonical Shh signaling

Shh transduces its signaling across the plasma membrane in the responding cells via either the canonical or non-canonical pathways. Canonical Shh signaling is the major pathway which has been studied intensively in vertebrate embryogenesis and tumorigenesis over the past few decades (Lum and Beachy, 2004). When secreted Shh ligand reaches its target cell, it binds with high affinity to the cell surface receptor, a twelve-pass transmembrane protein Patched-1 (Ptch1), to initiate the signal (Ingham, 2012). In the absence of Shh, Ptch1 normally represses the activity of Smoothened (Smo), the seven-pass transmembrane spanning receptor which is the major signaling component and switch in this pathway. The zinc-finger transcriptional factors of Glioma-associated oncogenes (Gli) family are inhibited by the cytoplasmic protein Suppressor of Fused (SuFu) (Tukachinsky et al., 2010). Conversely, in the presence of Shh binding to Ptch1, there is relief of the inhibition of Smo. Accumulated Smo receptor turns on a complex network leading to the regulation of three Gli transcriptional factors, the activators Gli1 and Gli2 and the repressor Gli3. Activated Gli accumulates in the nucleus and then controls the transcription of target genes, such as cyclin D1, cyclin E and N-Myc. Of note, besides acting as a transcriptional repressor mediated by a truncated fragment, Gli3 exists as a full length form which is able to activate transcription in specific systems (Dai et al., 2002).

In mammals, Ptch1 has a homologue Ptch2 that shares 54% sequence identity (Carpenter et al., 1998). All three mammalian hedgehog ligands bind both Ptch1 and Ptch2 receptors with similar affinity, making it difficult to discriminate the receptor specificity. However, Ptch2 appears to have a distinct downstream signaling role from Ptch1, as it is expressed at much higher levels in specific organs such as testis and ovary (O’Hara et al., 2011). In the absence of ligand binding, Ptch2 has a decreased ability to inhibit the activity of Smo, compared with Ptch1 (Alfaro et al., 2014; Zhulyn et al., 2015). Structurally, Ptch protein has a sterol sensing domain (SSD), which is essential for inhibiting the activity of Smo (Lindstrom et al., 2006). Normally, Ptch acts as a sterol pump and removes oxysterols produced by 7-dehydrocholesterol reductase. Upon binding of Shh protein or a mutation in the SSD of Ptch, the pump is turned off, which allows oxysterols to accumulate around Smo. This accumulation of sterols renders Smo to become active or stay on the membrane for a longer period of time (Reichrath and Reichrath, 2013). In contrast to *Drosophila*, vertebrates possess another level of hedgehog regulation through ligand-dependent antagonism mediated by Hh-interacting protein 1 (Hhip1). Hhip and Smo colocalize at the cell surface, Hhip has no effect on activity of Smo but modulates Smo localization. In addition, Hhip1 also sequesters hedgehog ligands (Ochi et al., 2006; Zhao et al., 2014).

### Non-canonical Shh signaling

It is increasingly recognized that not all Shh signaling proceeds through Gli protein activation, thanks to the studies in the fields of cancer biology, neurological disorders and metabolic diseases (Aberger et al., 2012; Jenkins, 2009; Teperino et al., 2014; Zhao et al., 2010). The definition of “non-canonical Shh signaling” refers to the cellular responses to Shh ligand that are independent of the activation of the Gli family of transcription factors. At least two separate non-canonical Shh signaling pathways are characterized: one is through Ptch1 but is unrelated to its inhibition on Smo; another is through Smo but is beyond Gli regulation (Figure 1C) (Brennan et al., 2012).

In the non-canonical Shh signaling engaged by Ptch1, Ptch1 is shown to independently induce human embryonic kidney 293T cells apoptosis provoked by Shh deprivation (Thibert et al., 2003). However, overexpression of Smo could not protect against cell death. Structurally, the C-terminal cytoplasmic domain of Ptch1 is a substrate for caspase-3, -7, and -8. Cleavage of Ptch1 is critical for apoptosis because mutation in the caspase site abolishes Ptch1-induced cell death. In addition, Ptch1 undergoes proteolytic processing at the C-terminus and the soluble C-terminal domain (CTD) translocates to the nucleus and mediates a new form of signal transduction (Kagawa et al., 2011). Different from *Drosophila* (Johnson et al., 2000), the CTD is not required for the canonical signal transduction but most likely has a distinct function in apoptosis and/or proliferation in mammals (Makino et al., 2001; Nieuwenhuis et al., 2007; Thibert et al., 2003). Besides apoptosis, exogenous Ptch1 in 293T cells induces the redistribution of cyclin B1 from the nucleus to the cytoplasm, resulting in reduced cell proliferation. This effect could be disrupted in the presence of Shh.

Another non-canonical Shh signaling mainly controls endothelial cells cytoskeletal reorganization and fibroblast migration through activating small GTPases and regulating fluctuations of calcium ions upon stimulation of Shh (Chinchilla et al., 2010; El-Zaatari et al., 2010; Polizio et al., 2011). It has been demonstrated that none of the three hedgehog ligands are able to induce Gli-target genes in human umbilical vein (HUVeC) or human cardiac microvascular endothelial cells (HMVeC), indicating that endothelial cells do not respond to hedgehog through the canonical pathway. However, all three hedgehog proteins promote endothelial cell tubulogenesis in 3D cultures in a Smo-dependent manner (Polizio et al., 2011). Consistent with the required cytoskeletal rearrangement for tubulogenesis, Shh, Ihh and Dhh all stimulate the small GTPase RhoA and actin stress fibers formation (Chinchilla et al., 2010). This effect, mediated by Smo and G proteins, defines a new non-canonical hedgehog pathway. In this system, stimulation by hedgehog ligands results in the formation of actin stress fibers within minutes, thus suggesting a non-canonical pathway based on the time course and the lack of detectable Gli-dependent transcription. Reminiscent of cytoskeleton remodeling, Shh could activate non-canonical signaling pathway to control axon guidance by stimulating the activity of Src family kinase (Yam et al., 2009).

## SHH SIGNALING AND CKD

### Shh induction and localization in CKD

Shh is known to be expressed in epithelial cells during development of several organs including kidney. In adult kidneys, however, Shh expression is largely silenced. Re-activation of Shh signaling has been implicated in the pathogenesis of tissue fibrosis in many organs including kidney, lung and liver (Chung et al., 2016; Cigna et al., 2012; Zhou et al., 2014).

As the major constituent of renal parenchyma, kidney tubular epithelium is the epicenter of various toxic, ischemic, metabolic and immunologic injuries (Li et al., 2012; Zhou et al., 2012). Although the damaged tubular cells may undergo a variety of maladaptive changes such as partial epithelial-mesenchymal transition (EMT), cell cycle arrest, defects in cell metabolism (Grande et al., 2015; Kang et al., 2015; Liu, 2010; Lovisa et al., 2015), one common consequence of these diverse responses is converting to a secretory phenotype (Zhou and Liu, 2016). Indeed, marked induction of Shh protein is observed in the fibrotic kidneys in all commonly used CKD models, including folic acid (FA), unilateral ureteral obstruction (UUO), ischemia reperfusion injury (IRI), adriamycin (ADR) and 5/6 nephrectomy, although it is barely detectable in normal kidneys (Ding et al., 2012; Fabian et al., 2012; Rauhauser et al., 2015; Zhou et al., 2014). In human biopsy specimens, Shh is also specifically induced in renal tubular epithelium of the diseased kidney, regardless of the initial etiologies (Zhou et al., 2014). Meanwhile, the sensor receptor Ptch1 and transcriptional factor Gli1, Gli2 are also upregulated in high-grade fibrotic human kidneys (Kramann et al., 2015; Zhou et al., 2014). These results suggest that Shh upregulation is a common pathologic finding in a wide variety of kidney diseases.

**Figure 2:**
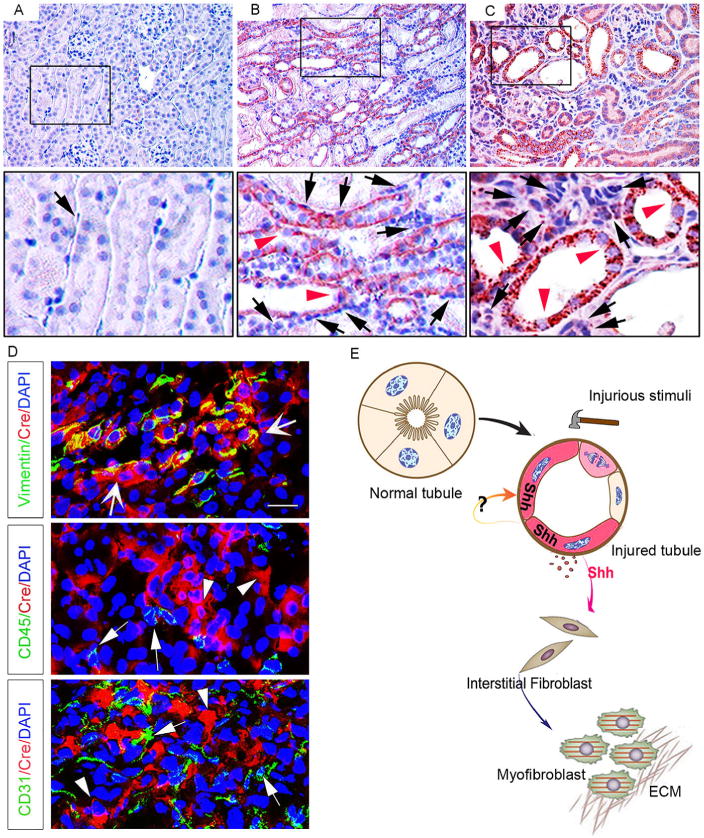
Tubule-derived Shh targets interstitial fibroblasts. A–C, Representative micrographs of immunohistochemical staining show marked induction of Shh protein after renal ischemia reperfusion injury (IRI) at 1 (B) and 10 days (C), respectively. Shh is barely detectable in sham control (A). At 1 day after IRI, Shh expression was significantly induced (B, red arrowhead) in renal tubules. At 10 days, sustained activation of Shh in renal tubules was observed (C, red arrowhead). D, Identification of the interstitial fibroblasts as Shh-responsive cells in fibrotic kidneys. Transgenic Gli1-CreER^T2^ mice were subjected to IRI for 10 days, and kidneys subjected to double immunostaining for Cre recombinase (red) and various cell type-specific markers (green). Wide arrows indicate cells with positive staining for both vimentin and Cre; arrowheads denote Cre-positive cells; arrows show CD45- or CD31-positive cells. Panel D was reproduced from our published paper (Zhou et al., 2014) with permission. E, Schematic diagram shows that Shh mediates epithelial-mesenchymal communication between injured tubules and interstitial fibroblasts, leading to fibroblast activation and proliferation, as well as matrix overproduction.

Shh induction is predominantly localized in renal tubular epithelium (Figure 2), whereas interstitial cells in the expanded interstitium are largely negative for Shh staining. Most importantly, upregulation of Shh protein is an extremely early event in different models *in vivo*, suggesting a potential role for Shh in triggering the first wave of defense mechanism after tissue damage. *In vitro*, a variety of pathologic cues such as TGF-β1 and Wnt induces Shh mRNA expression and protein secretion by tubular epithelial cells. Based on these observations, we recently propose that Shh is a novel, inducible, tubule-derived growth factor that mediates epithelial-mesenchymal communication (EMC) after kidney injury (Ding et al., 2012; Zhou et al., 2014).

### Shh promotes kidney fibrosis

The functionality of Shh in the development and progression of CKD has been extensively studied by both gain- and loss-of function approaches. Emerging evidence indicates that along with the progression of kidney injuries, hyperactive Shh signaling causes fibroblast activation, proliferation and matrix over-production by multiple mechanisms, primarily via a mode of epithelial-mesenchymal interaction.

In mouse model of IRI, overexpression of Shh transgene through a hydrodynamic-based gene transfer approach promoted fibroblast proliferation and matrix overproduction and deposition, ultimately leading to an increased kidney fibrosis and aggravated kidney dysfunction (Zhou et al., 2014). The population of Shh-responsive Gli1-positive cells is markedly expanded in renal interstitium after Shh transgene expression. *In vitro*, human recombinant Shh protein activated Ptch1 and Gli1 and induced α-smooth muscle actin (α-SMA), desmin, Snail1, fibronectin, and collagen I expression (Ding et al., 2012; Zhou et al., 2014), suggesting a critical role for Shh signaling in regulating myofibroblast activation and matrix production. Shh also promotes cultured rat kidney fibroblast proliferation and stimulates the induction of numerous proliferation-related genes (Zhou et al., 2014). Shh ligand also triggers pericyte-like cell proliferation *in vitro* (Fabian et al., 2012), suggesting a role for this pathway in regulating cell cycle progression of myofibroblast progenitors during the development of renal fibrosis.

Using conditional knockout mouse model, we recently show that tubule-specific deletion of Shh reduces fibrotic lesions after kidney injury (data not shown), suggesting that Shh not only is sufficient but also necessary for fibrosis development in the kidney. This conclusion is also supported by genetic and pharmacologic manipulations of the Shh downstream mediators Smo or Gli proteins. It is shown that the kidneys in Gli1-deficient mice are protected against the development of tubulointerstitial fibrosis after UUO (Ding et al., 2012; Rauhauser et al., 2015). Smo inhibitors such as cyclopamine also repress the induction of Gli1, Snail1 and α-SMA, and reduce matrix expression and mitigate fibrotic lesions in obstructive nephropathy (Ding et al., 2012; Rauhauser et al., 2015), although it does not affect renal Shh expression. Another study demonstrates that Gli2 in Shh pathway is expressed in myofibroblast progenitors, and drive cell-cycle progression in cultured mesenchymal stem cell-like progenitors (Kramann et al., 2015). Myofibroblast-specific deletion of Gli2 or overexpression of Gli3 repressor attenuates renal fibrosis after UUO (Kramann et al., 2015). In addition, suppression of Shh signaling is also associated with decreased macrophage infiltration after obstructive injury (Rauhauser et al., 2015).

## TARGETS AND MECHANISM OF SHH SIGNALING IN KIDNEY FIBROSIS

### Fibroblasts as a major target of canonical Shh signaling

Given that Shh is a secreted protein mainly produced by renal tubular epithelium, an important question that needs to be clarified is the cellular targets of Shh in diseased kidney. To address this issue, three independent groups utilized the Gli1^lacZ^ mutant mice, the so-called hedgehog-reporter mice, which harbor a β-galactosidase (β-Gal) “knock-in” mutation. Under the control of the native Gli1 upstream promoter/enhancer elements, *lacZ* expression in these mice authentically recapitulates the expression pattern of the endogenous Gli1 mRNA. Results show that interstitial fibroblasts, including platelet-derived growth factor receptor (PDGFR)-β-positive pericytes, are identified as principal Shh-responding cells *in vivo* in UUO and IRI models (Ding et al., 2012; Fabian et al., 2012; Rauhauser et al., 2015; Zhou et al., 2014). Further studies using Gli1-Cre mice have demonstrated that the Shh-responding cell population does not include CD31-positive endothelial cells and CD45-positive monocytes in renal interstitium (Zhou et al., 2014) (Figure 2D). These findings are consistent with the observations that cultured kidney fibroblasts respond to Shh stimulation *in vitro*, leading to an enhanced cell proliferation and myofibroblastic activation. Intriguingly, Shh had no effect on the proliferation of renal tubular epithelial cells (Zhou et al., 2014). Therefore, as a tubule-derived growth factor, Shh specifically targets interstitial fibroblasts and mediates epithelial-mesenchymal communication via a paracrine fashion (Figure 2).

It should be pointed out that Gli1 expression only reflects the activation of the canonical hedgehog signaling in the responding cells. In this regard, possibility exists that other types of cells in the kidney besides interstitial fibroblasts, such as tubular epithelial cells, endothelial cells and infiltrated inflammatory cells, may also respond to Shh via Gli-independent, non-canonical pathway. More studies are warranted in this area to define the role and mechanism of Shh in regulating the activities of many other kidney cells under pathologic conditions.

### Crosstalk with other signaling pathways in kidney fibrosis

Increasing evidence suggests that Shh signal interacts with several key signaling pathways and operates in a coordinated fashion during renal fibrogenesis. For instance, besides Shh signaling pathway, TGF-β1, Wnt/β-catenin and Notch pathways are well recognized to play crucial roles in the evolution of renal fibrosis in CKD (Bielesz et al., 2010; He and Dai, 2015; He et al., 2009; Tan et al., 2014; Zhou et al., 2016). However, the role of Shh signaling is quite unique in this process in that it specifically targets interstitial cells, while it is mainly produced by the injured tubules. In this way, Shh acts as a master communicator between the injured tubular epithelial cells and interstitial fibroblasts, leading to fibroblast activation and expansion, which plays a central role in renal fibrogenesis (Fabian et al., 2012; Kramann et al., 2015; Zhou et al., 2014). In contrast, the major targets of Wnt and Notch signals are tubular epithelial cells, which then indirectly promote kidney fibrosis through the processes such as partial EMT (Liu, 2006, 2010).

Over the past several years, evidence of crosstalk among Shh, Wnt, Notch, TGF-β1 and other signaling pathways has been frequently reported in renal fibrosis. For example, Shh is known to upregulate Wnt2b and Wnt5a (Katoh and Katoh, 2009), as well as Notch ligand Jaggad2 (Katoh and Katoh, 2008). Wnts could induce Shh expression in epithelial cells, and both Wnt and Shh signaling can induce Snail1 expression in fibrotic kidneys (Ding et al., 2012; Wang et al., 2011; Zhou and Liu, 2015). Gli1 could induce Snail1 and promote β-catenin nuclear translocation in epithelial cells (Li et al., 2007). TGF-β1 is reported to induce several key components of the Wnt, Shh and Notch signaling pathways (Bielesz et al., 2010; Wang et al., 2011). Therefore, crosstalk between these signal pathways has the potential to orchestrate complex cellular responses to extracellular stimuli. These findings present new challenges—as well as opportunities—for discovering novel interventional strategies.

## STRATEGIES TO TARGET SHH SIGNALING IN KIDNEY FIBROSIS

**Figure 3:**
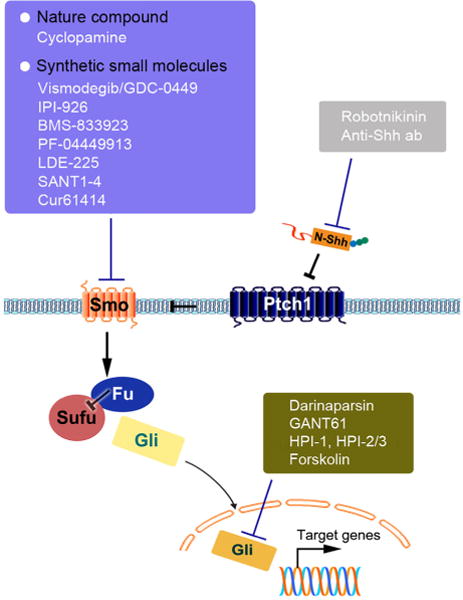
Therapeutic strategies to target Shh signaling pathway. The Shh signaling could be blocked at different levels, including Shh ligand inhibitors, Smo receptor inhibitors and transcriptional factors Gli inhibitors. Among these antagonists, Smo inhibitors (cyclopamine and IPI-926) and Gli inhibitors (Darinaparsin and GANT61) are tested in the setting of fibrotic CKD. Except for IPI-926 (Fabian et al., 2012), all Shh signaling inhibitors are able to attenuate renal fibrosis in experimental models.

In view of the central role of Shh signaling in renal fibrosis, targeting this pathway might be an effective strategy to halt and even to reverse the progression of CKD. In theory, the Shh signaling could be blocked at different levels along the signal transduction route. Recently published literature describes small molecule inhibitors in Shh pathway as the following categories (Gonnissen et al., 2015; Ruat, 2015): inhibitors targeting Smo; strategies to target the upstream N-Shh ligand; and inhibitors targeting the downstream transcription factors Gli1 and Gli2 (Figure 3). The translation of these exciting new pharmacologic therapies to the treatment of kidney diseases has just begun in the preclinical setting (Table 1). In this review, we will focus on the Smo inhibitors and Gli inhibitors, which have been recently assessed for their efficacy in the treatment of kidney diseases.

### Smo inhibitors

The most common method to target Shh pathway is through manipulation of Smo activity because it is the main signal transducer of this pathway. Smo is responsible for a large variety of developmental processes. It belongs to class F (Frizzled family) of the G-protein-coupled receptor (GPCR) superfamily (Ruiz-Gomez et al., 2007). Recently, robust clinical investigations implicate Smo as a novel therapeutic target in human cancers.

As the first member of a class of small molecular compounds that specifically inhibit the activity of Smo, cyclopamine (CPN) has been utilized in the treatment of experimental renal fibrosis (Ding et al., 2012; Rauhauser et al., 2015). CPN is a natural alkaloid derivative isolated from a plant of the corn lily family (Incardona et al., 1998; Strand et al., 2011). In renal fibrogenesis, targeting Smo with CPN inhibits fibroblast activation and matrix production *in vitro*, and it reduces matrix expression and mitigates fibrotic lesions *in vivo* after UUO and IRI (Ding et al., 2012; Zhou et al., 2014). Meanwhile, induction of the downstream target of Smo, Gli1 and Snail1, are largely abolished by CPN (Ding et al., 2012; Zhou et al., 2014). However, it does not affect upstream Shh ligand expression. The shortcomings of CPN are its short half-life and off-target effects at higher doses (Lipinski et al., 2008; Zhao et al., 2009). Therefore, a more soluble and potent CPN derivative, IPI-926 (Saridegib), has been explored in clinical trials for basal cell carcinoma (BCC) and metastatic pancreatic cancer. After UUO, IPI-926 abolishes Gli1 induction, but not Gli2 *in vivo*, suggesting the existence of Smo-independent Gli activation in the model. In contrast to CPN, IPI-926 did not attenuate renal fibrosis (Fabian et al., 2012). These differences might be due to the chemical diversity of Smo modulators and their different pharmacological properties, because there are at least two well-characterized ligand-binding pockets presented in Smo receptor: one in the extracellular domain (ECD), and one in the TM domain.

The discovery of CPN and its derivative has heralded extensive research for development of Smo antagonist. A major breakthrough is the recent approval of a novel Smo antagonist Erivedge/Vismodegib (GDC-0449) in the Shh field by the FDA for treating metastatic BCC and locally advanced BCC deemed untreatable by surgery or radiation (Robarge et al., 2009). However, its effects in kidney diseases remain to be investigated and validated in the preclinical setting before considering any clinic trials.

### Gli inhibitors

Although Smo has been considered as the most common target of Shh signaling, the importance of the downstream regulators of Smo, such as SuFu and Gli1/2 cannot be ignored. After all, Smo antagonists do not repress upstream ligands expression, whereas a compound directed against the downstream targets would be able to more effectively inhibit the entire pathway. In this regard, Darinaparsin, a novel organic arsenical with optimized pharmacokinetic properties, is currently in clinical studies for hematologic malignancies and solid tumors. As the antagonist of Gli proteins, Darinaparsin directly targets Gli2 protein and induces Gli-dependent cell-cycle arrest in renal myofibroblasts, thereby preventing myofibroblast proliferation and ameliorate kidney fibrosis *in vivo* (Kramann et al., 2015). Besides Darinaparsin, GANT61, a small-molecule inhibitor of Gli, also displayed the ability of ameliorating renal fibrosis in mice, even it is administered after injury (Kramann et al., 2015).

## SHH SIGNALING AND THE FIBROTIC DISEASE OF OTHER ORGANS

Besides kidney, increasing evidence suggests that activation of Shh signaling is also associated with tissue fibrosis in other organs including lung and liver. *In vitro*, it has been well documented that recombinant Shh protein induces an activated phenotype of cultured lung fibroblasts and hepatic stellate cells, as manifested by an augmented cell proliferation, migration, excessive ECM production (Hu et al., 2015; Yang et al., 2008). Shh signaling is upregulated in animal models and patients with idiopathic pulmonary fibrosis (IPF), other interstitial lung diseases and nonalcoholic fatty liver disease (NAFLD), with nuclear accumulation of Gli1, Gli2 in the fibrotic areas and an increased expression of the Shh downstream target genes. In lung, Shh is predominantly expressed in type II like epithelial cells of terminal bronchioles and alveoli (Bolanos et al., 2012). Shh activation causes aberrant epithelium-fibroblast interactions and directly triggers pulmonary fibrosis after injury (Hu et al., 2015; McGowan and McCoy, 2013). Unlike lung, Shh is found in both ballooned hepatocyte and hepatic stellate cells in the liver of NAFLD, which eventually leads to liver fibrosis or cirrhosis (Hirsova and Gores, 2015; Rangwala et al., 2011). By using hydrodynamics-based gene transfer approach, hepatic expression of Shh induces liver fibrosis, which is accompanied by concurrent activation of hepatic stellate cells, and upregulation of various fibrogenic genes (Chung et al., 2016). Meanwhile, hepatic stellate cells could produce Shh through an autocrine fashion to accelerate progression of liver diseases. In addition, activation of Shh signaling is also responsible for dermal fibrosis in systemic sclerosis (Horn et al., 2012). Therefore, activation of Shh signaling perhaps is a generalized mechanism leading to tissue fibrosis after injury.

## SUMMARY

As a potent morphogen, Shh controls cell proliferation, differentiation and morphogenesis in embryonic kidney (Yu et al., 2002). In the past few years, increasing evidence indicates that re-activation of Shh signaling plays a critical role in the onset and progression of kidney fibrosis after various insults. Shh protein is produced and secreted by the injured tubules but specifically targets interstitial fibroblasts, leading to their activation, proliferation and expansion. Through such a paracrine fashion, Shh acts as a master communicator and mediates epithelial-mesenchymal communication (EMC) during renal fibrogenesis. By characterizing the receptors, downstream regulators and transcriptional effectors in the Shh signaling, pharmacologic manipulation of this pathway is now being attempted in a variety of animal models of CKD in preclinical setting.

Our knowledge of Shh signaling remains in its infancy in the field of nephrology. Continuous exploration of renal pathogenesis linked with the Shh pathway and validation of pharmacological intervention strategies is an urgent task for researchers and nephrologists. There are many questions to be answered. For instance, since upregulation of Shh is an early event after injuries, what is the function of Shh in the setting of acute kidney injury? What is the role of Shh in glomerular diseases where the specialized epithelium (podocytes) communicates with mesangial cells? What is the extent of interaction between Shh and other pathologic signal pathways? It will be of great interest to see these different aspects of Shh signaling unveiled in the near future.

## Figures and Tables

**Table 1:** The strategies to target Shh signaling in fibrotic CKDa)

Disease model	producing-cells	Responding cells	Target/inhibitor	Outcome	References
UUO	Tubular cells	Fibroblast	Smo/cyclopamine	Improved	(Ding et al., 2012)
IRI	Tubular cells	Fibroblast	Smo/cyclopamine	Improved	(Zhou et al., 2014)
UUO	Tubular cells	Pericyte/fibroblast	Smo/IPI-926	Negative	(Fabian et al., 2012)
UUO	Tubular cells		Gli2/darinaparsin	Improved	(Kramann et al., 2015)
UUO	Tubular cells	Pericyte/macrophage	Smo/cyclopamine	Improved	(Rauhauser et al., 2015)

a) UUO, unilateral ureteral obstruction. IRI, ischemia reperfusion injury
